# Malaysian Child Restraint Issues: A Brief Narrative Review

**DOI:** 10.3390/ijerph17061922

**Published:** 2020-03-16

**Authors:** Roszalina Ramli, Siti Salmiah Mohd Yunus

**Affiliations:** Department of Oral and Maxillofacial Surgery, Faculty of Dentistry, Universiti Kebangsaan Malaysia, Kuala Lumpur 50300, Malaysia; sitisalmiahmy@gmail.com

**Keywords:** Malaysia, child restraint law, child restraint system, unrestrained injuries

## Abstract

The child restraint legislation in Malaysia becomes mandatory from 1 January 2020. Prior to commencement of the rule, a survey showed that only 36% of Malaysian parents were aware of the importance of a child restraint system (CRS) and only 27% usage was reported during travel. The Malaysian Institute of Road Safety report showed that children transported in private vehicles were the leading groups of casualties among children aged 1 to 4 years old (43.8%) and 5 to 9 years old (30.2%), respectively. We performed a narrative review using the PubMed, ScienceDirect and Google Scholar databases using keywords such as child restraint system, unrestrained injuries, Malaysia and epidemiology. The objectives of this review were: (1) to determine the prevalence on the use of CRS in Malaysia, (2) to evaluate the injuries related to unrestrained children and (3) to show the nation’s preparation towards implementation of the child restraint law. Six papers on prevalence, one paper on injury and six mainstream newspaper were included in this study. The prevalence of a CRS use was shown between 5% to 41.8%. In relation to injury, the only publication from this country showed that among 19 children involved in a car crash, five (26.3%) children had non-craniomaxillofacial (CMF) injuries, ten (52.6%) with CMF injuries only, two (10.5%) with both CMF and non-CMF injuries and two (10.5%) without any injury. Overall, the Injury Severity Score (ISS) range was between 0 to 13 (median, 1.00; interquartile range, 1). Preparation to comply with the best practice of the child restraint law is still ongoing, especially those addressing the issues related to the low-income parents in the country. Due to scarcity of publication and data on the CRS use and injuries related to its non-usage, it is advocated that parallel with the implementation legislation, vigorous forms of public education as well as good data management must be performed and monitored regularly by the road safety authority in this country.

## 1. Introduction

The 2018 World Health Organization (WHO) Global Status Report on Road Safety reported road injuries as the leading cause of death for children and young adults aged 5 to 29 years [1]. Globally, car occupants contributed 29% to all road traffic deaths [2]. In Malaysia, children transported in private vehicles (car, van, four-wheel drive (4WD)) are the first and second leading groups of casualties among those aged 1 to 4 years old (43.8%) and 5 to 9 years old (30.2%), respectively [3]. For children in the age group of 0 to 4 years old, the increasing fatality rate is alarming, i.e., from 2.7 per 100,000 population in 2004, to 4.2 per 100,000 population in 2009. This shows a 55.5% increment over a five-year period. For children aged 5 to 9 and 10 to 14 years old, the fatality rate per 100,000 population plateaued from 2004 to 2006 and then showed a declined trend from 2007 to 2008. However, the trend increased again in 2009 [4].

To reduce road fatalities among children transported in the four-wheel vehicles, a child restraint system (CRS) is required. This is to ensure that the child occupant is firmly secured in his/her seat so that he/she will not be thrown against the car interior or ejected from the vehicle in the event of sudden braking or collision [5]. The restraint must absorb the kinetic energy resulted from the crash without itself injuring the child, distribute the energy load evenly to the bones rather than the soft tissues, limit the crash forces experienced by the vehicle occupant by prolonging the time of deceleration, and limit the contact of the occupant with the interior vehicle structures [6].

The best practice recommendation from the American Academy of Pediatrics is that all infants and toddlers must ride in a rear-facing CRS, until they reach the highest weight or height allowed [6]. The young child’s posterior torso, neck, head, and pelvis must be well-supported so that the crash energy could be distributed over the entire body in the event of crash. Developmental considerations, including incomplete vertebral ossification, more horizontally oriented spinal facet joints, and excessive ligamentous laxity, put young children at risk for head and spinal cord injury and a rear-facing CRS addresses this risk by supporting the child’s head, preventing the relatively large head from moving independently on the proportionately smaller neck [6]. When used correctly, child safety seats have been shown to reduce the risk of death of children aged between 2 to 6 years by 28% [7]. Another study showed that a child up to 4 years of age in a forward-facing CRS has a 50% lower risk of injury and in a rear-facing seat, an 80% lower risk [8]. This was compared to when an adult seatbelt was worn, where the injury reduction was only 32% [8]. For children aged 5 to 9 years, booster seats lessened the injury by 52%, while for seatbelts, the reduction was 19%, and for older children aged 10 to 14 years, the seatbelts cut down injury by 46% [8].

In general, there are very few publications on children and the CRS in this country. A similar trend was observed involving publications from the Western Pacific and South-East Asia regions.

The objective of this brief review was to determine the prevalence or proportion and awareness of CRS use in this country. In addition, published local data on injury related to non-usage (versus usage) of the CRS was investigated. Local news on the nation’s preparations towards implementation of the child restraint regulation on 1 January 2020 was also explored.

## 2. Methods

The following databases: PubMed, ScienceDirect and Google Scholar, were included in this narrative review. Keywords such as child restraint system, unrestrained injuries, Malaysia, and epidemiology were used. Apart from the above databases, the search was performed through the snowballing technique, exploring references from the initial search. World Health Organization publications such as the annual Global Status Report on Road Safety and others related to child injury, seatbelts, and child restraint modules were also included.

The databases were selected based on their coverage of the topic of interest, while for the local newspapers, the selection involved all mainstream newspapers. Duplication was removed by analyzing the content, a more detailed content was selected over brief reports. The focus for the newspaper search was to examine the preparation made by the government as well as responses from individuals or agencies ahead of child restraint regulation implementation.

The selection criteria included all types of study, i.e., randomized control trial or a clinical trial, cohort, case-control, cross-sectional, and retrospective record review studies. The search included publications in English and Malay language. The date of publication was not specified as the intention was to assess all reported work on child restraint or vice versa from as early as possible until 15 December 2019 (the final phase of this manuscript writing).

The interpretation criteria for the proportion of CRS usage was proposed as below:

High usage: >75%

Moderate: 50–75%

Low: <50%

Selected papers were imported into Mendeley Desktop 1.18 following assessment of the full paper.

### Methods of Review

The studies were divided into three categories: (1) prevalence on the use of CRS, (2) injury related to unrestrained children, and (3) the nation’s preparation towards receiving the child restraint law.

These categories were selected to serve as a baseline data for comparison later on, between post-implementation of the child restraint law with the pre-unrestrained era particularly related to injury prevention.

Following completion of extraction of data, a synopsis for each study was created to present the relevant information.

## 3. Results

Overall, the publication on child restraint in Malaysia is very limited. Six papers on prevalence and one paper on injury were included in this review. On the other hand, six mainstream newspapers were also included. The 2018 WHO Global Status Report on Road Safety [1] was used to show the child restraint status in the Association of Southeast Asian Nations (ASEAN) countries.

The search above yielded in findings that could be discussed under the following headings:

- Child restraint scenario in Malaysia.
- Child restraint law in Malaysia and the ASEAN countries.
- Pattern of injuries in unrestrained children in a selected center in Malaysia.
- Preparation prior to implementation of child restraint law.

### 3.1. Child Restraint Scenario in Malaysia

In general, all six papers showed low percentage of CRS or seatbelt usage among Malaysian children.

A roadside observation study showed a low percentage of seatbelt wearing among children: front seat, 11.8%, and rear seat, 5.8% [9]. This study observed occupants in vehicles, comprising 7247 cars, vans, and taxis at selected locations in Klang Valley, Malaysia.

Another study which observed children transported to daycare centers in a suburban district in the state of Selangor, Malaysia, showed that of 537 children, only 9.5% were on the CRS. Among these children, 13% were front seat passengers, while another 5% sat at the rear [10]. Among the restrained children, 22% and 7% were driven to the daycare centers by belted and unbelted drivers, respectively. 15% children were restrained by female drivers compared to 5% by male drivers.

A cross-sectional study conducted in Malacca, Malaysia, in 2004, showed that only 27.4% drivers used child safety seats. Three drivers’ characteristics were found to be significantly attributed to the CRS usage: age less than 40 years, tertiary education level, and had positive attitude [11].

Presence of an airbag on the front passenger seat does not necessarily indicate that the child passenger would be fully restrained. A study on the association between vehicles with airbags and CRS showed that 11.2% of children seated on the front seats were restrained, while in the vehicles without, 17.1% were restrained. The presence of front passenger airbags did not have a significant association with the use of a CRS. However, there was a significant relationship between belted drivers and restrained children where children were four times more likely to be restrained if the drivers were belted compared to those who were not [12].

In addition, in a study observing the use and misuse of the CRS, the reasons provided by the respondents for not restraining their children were: (1) children were perceived as big enough or “grown up” and did not require a CRS (23.2%), (2) children’s refusal to be restrained (13.9%), (3) car does not have a CRS (6.0%), (4) short distance travel (4.2%), and others [13]. A total of 178 parents were interviewed and 267 children were observed. In this study, 11.6% of the children were restrained using rear facing CRS, 19.1% were in front facing CRS, 4.5% used booster seats, and 7.9% used adult seatbelts, 34.8% of the children were seated in the front passenger seat while the rest were in the second-row seats (mostly behind front passenger seat).

### 3.2. Child Restraint Law in Malaysia and the ASEAN Countries

Malaysia and its close neighbors are known as the Association of Southeast Asian Nations, or ASEAN. ASEAN countries are grouped differently by the WHO, i.e., the South East Asia and Western Pacific regions. ASEAN countries have many similarities in relation to road transportation, safety issues, and road crashes. To date, of the ten countries, only Singapore, Brunei Darussalam, Cambodia, and Lao People’s Democratic Republic have a child restraint law (Table 1). Beginning 1 January 2020, Malaysia joins these four countries with a new child restraint rule.

In relation to front seat child passenger, the Phillipines and Cambodia specified a certain age while Singapore allows children sitting in the front seat, provided a child restraint is present.

### 3.3. Pattern of Injuries in Unrestrained Children in a Selected Center in Malaysia

Only one study was found focusing on injuries among restrained and unrestrained children in one center in Malaysia. Yunus et al. showed that among 19 Malaysian child passengers aged between 0 to 13 years old (mean age 6.02 years; standard deviation (SD), 3.46), three were with seatbelts and one was in the child safety seat [15]. This retrospective study looked at the occurrence of craniomaxillofacial (CMF) injuries among restrained and non-restrained children. Among the 19 children, five (26.3%) children had non-CMF injuries, ten (52.6%) with CMF injuries only, two (10.5%) were with both CMF and non-CMF injuries, and two (10.5%) were without any injury [15]. The most severe head injury sustained in a restrained child was a base of skull fracture, while for an unrestrained child, a cerebral concussion [15].

Among the children with facial injury, 22% and 50% in restraint systems sustained middle and lower face injuries, respectively. In addition, in children without a restraint, 100% sustained upper face soft tissue injuries, 77.8% with middle face soft tissue injury, and 50% with lower face injuries. Overall, the Injury Severity Score (ISS) range was between 0 to 13 (median, 1.00; interquartile range (IQR), 1) [15].

### 3.4. Preparation Prior to Implementation of Child Restraint Law

Child restraint system use is made compulsory to all private vehicles beginning from 1 January 2020. This move is welcomed by many road safety agencies and non-governmental organizations as well as individuals [16,17]. The Safe Kids Malaysia Universiti Putra Malaysia, which is an affiliate of Safe Kids Worldwide, emphasized on law enforcement, and that enforcement should be regarded as part of education [16].

No penalty or summon should be issued by the Road Safety Department during the first six months following commencement of the law and this was emphasized by the Minister of Transport of Malaysia [18]. The focus at the early phase is to educate the public instead of punishing them [18].

The Malaysian Institute of Road Safety Research (MIROS) plays a major role in making sure the enforcement is well understood by the public. This includes:

(i) Educating the public through live demonstration of the dynamics of the child seat and others.
(ii) Producing a guideline on child safety seats specification, types, and placement according to the vehicle seats and number of children.

Only child seats that meet the UNECE R44 and R129 design standards are allowed for sale in Malaysia [19,20]. This is in line with the World Health Organization’s recommendation [1]. The guidelines specify four different types of seats according to the child’s height and weight [19,20]:

(i) Type 1: from birth up to 13 kg (up to a height of 83 cm, approximately 0 to 18 months),
(ii) Type 2: 9–18 kg (71 cm and above, approximately 15 months to four years),
(iii) Type 3: 15–25 kg (100 cm and above, approximately four to seven years), and
(iv) Type 4: 22–36 kg (up to 135 cm, approximately six to 12 years).

Despite all preparations made to implement the legislation, issues and concerns from the B40 community (Bottom 40%, as in the Malaysian income classification by the Department of Statistics) have been raised and acknowledged by the Malaysian government. In 2019, the B40 group earned a median income of Ringgit Malaysia (RM) 3000 (equivalent to USD 733.05)). Modification to the child restraint rule at this early stage includes exempting large families with small four-wheel vehicles from installing the child restraint seats [21]. Reducing the import duty and minimizing the tax of the child seats to reduce the burden of the B40 group have been included. Concurrently, the Domestic Trade and Consumer Affairs Ministry will closely monitor the online sale of CRS, to ensure they comply with the stipulated standards [20]. The online purchase of the CRS is significantly cheaper however the standard may be compromised.

To ease the burden of the B40 or the low-income parents further, groups like the BMW Malaysia with its partner, Childline Foundation, offer subsidized seats at an affordable price, i.e., RM 100 (equivalent to USD 24.45) [22].

## 4. Discussion

A recent report from one of the mainstream media showed that awareness of Malaysian parents on the importance of using child safety seats was only at 36% [18]. This was in accordance with the studies discussed in this review. Generally, low CRS usage, i.e., between 5% to 41.8%, was documented [9,10,11,12,13]. Preparation towards implementation of making CRS mandatory was described in the 3.4. Factors described by Kulanthayan et al. [11] and the socio-economic status of the families must be explored in depth for effective strategic planning. Measures to educate the public on the CRS use and road safety must be carried out continuously to address all walks of life, from the classic advertisements in the newspapers, television, and radio to popular social media and sites [23]. Apart from education, appropriate advocacy, legislation, and enforcement are necessary to increase CRS use among the public [11].

In relation to injury literature, local papers describing injuries related to use or non-use of CRS is very scarce. Only one article was found for the assessment of injury. For comparison and validation, injury papers from the ASEAN countries were searched, however it was unsuccessful. Eventually, papers published elsewhere were considered even though they were not comparable in terms of geography, socio-economy, mechanism of injury, and others. The findings in these papers served as a reference to this review.

It has been shown that unrestrained children and those seated in the front seats have higher risk of sustaining severe injuries, and this results in longer hospitalization and higher costs of trauma care compared to properly seated and restrained children [24,25].

Chan et al. showed significant occurrence of intracranial, intrathoracic, and intraabdominal injuries in the unrestrained children [24]. Al-Jazaeri et al. showed that overall, more back-seat unrestrained children sustained injuries compared to the front seat passengers [26]. Front seat children were shown to sustain more head, neck, or facial injuries compared to back-seat children with pelvic or long bone injury [26].

A 1998 article utilizing the National Automotive Sampling System (NASS) database showed a pattern of injuries in the absence of restraint use in 23–43% of the children [27]. This article may be used as the reference for pattern of injuries as the CRS non-use was similar. This study showed that for children aged less than one year old, 60% of them sustained a facial injury [27]. Head injuries comprised 10% of the total injuries, however, the form of injuries was more severe. Abdominal and lower extremity injuries started to appear frequently in older children, aged between one to four years old. For children aged five to ten years old, spinal injuries became more prominent. By the age of 11 to 16 years, injuries to the spine, upper, and lower extremities became more common than injuries to the face and head. However, most of the severe injuries, i.e., Abbreviated Injury Scale (AIS) 3 to 5 still involved the head and abdomen [27].

Table 2 shows the pattern of injuries involving unrestrained children. Malaysian data is shown alongside the studies from Saudi Arabia and the United States of America [15,24,26].

As described in the Results Section, the method of implementation of the child restraint law is more educational in the first six months rather than punitive. Issues such as unaffordability among the low social income group, large families with small vehicles, and others are being considered by the Malaysian government. These issues and others that may surface during this six-month educational period should be managed thoroughly prior to the full-fledged implementation of the law. The law will be assessed using the following four best practice criteria [1]:

(1) Presence of a national child restraint law,
(2) Requirement for a child to use a child restraint, at least until ten years of age or 135 cm height,
(3) Restrictions for children under a certain age or height to sit in the front seat, and
(4) Reference to or specification of a standard for child restraints.

## 5. Limitation

The main limitation of this review is the literature on this topic in this country is very scarce, particularly on injuries among the unrestrained children. This will make comparison with the injured children post-legislation in the future difficult. The national road crash statistics published by Royal Malaysia Police annually is also lacking in the description of restrained versus unrestrained children.

## 6. Conclusions

The new road safety law on child restraint use in Malaysia was made effective beginning from 1 January 2020. It is compulsory for Malaysian children under the age of 13 years old to be in a standard child restraint system during travelling in a private vehicle. At present, the statistics on usage of a child restraint system is very low and the data on injuries among unrestrained children is very scarce. As a middle-income country with issues of unaffordability and number of passenger-size of a car discrepancy, the intention to get moderate usage (50% to 75%) of the CRS may take more than a decade. Smart purchasing strategies with subsidies as well as effective enforcement and vigorous road safety education focusing on injuries are among the strategies that could be adopted to save more children on Malaysian roads.

## Figures and Tables

**Table 1:** Child Restraints in the Association of Southeast Asian Nations (ASEAN) Countries [1,14].

Country	Child Restraint Law		Child Seated in Front Seat
	Yes, since	No	
Malaysia	✓ Beginning from 1 January 2020		-
Singapore	✓		Allowed in a child restraint
Indonesia		✓	No restriction
Brunei Darussalam	✓		-
Thailand		✓	No restriction
Phillipines		✓	Prohibited under 6 years old
Vietnam		✓	No restriction
Myanmar		✓	No restriction
Cambodia	✓		Prohibited under 10 years
Lao People’s Democratic Republic	✓		No restriction

**Table 2:** Injuries sustained by restrained versus unrestrained children.

Authors; Publication Year; Country	Age of the Children, Range, Mean	Type of Study	Sample Size	Injuries	
				Restrained	Unrestrained
Yunus et al.; 2015; Malaysia [15]	0 to 13 years old	Cross sectional study: (1) Retrospective record review (2) Telephone interview	Restrained: 4 (1 front seat, 3 back seat) Unrestrained: 15	This study focused on head and facial injuries predominantly.	
				Head injury: 1 child with base of skull fracture	Head injury: 1 child with cerebral concussion
				Facial injury: 22% with midface injuries 50% with lower face injuries	Facial injury: 100% with upper face injuries 77.8% with midface injuries 50% with lower face injuries
Chan et al.; 2006; United States [24]	14 years old and younger	Retrospective chart review	Restrained: 255 Unrestrained: 81	Odds between unrestrained and restrained pediatric victims: (1) Hospital admission: 14.48 (CI 5.91, 38.63) (2) Intraabdominal injuries: 20.16 (CI 2.36, 930.68) (3) Intrathoracic injuries: 13.09 (CI 1.26, 647.05) (4) Intracranial injuries: - (9 unrestrained sustained this injury compared to 0 restrained) (5) Fractures: 5.85 (CI 2.13, 16.89) (6) Need for surgery: 13.09 (CI 3.30, 74.33) (7) Need for blood products: 27.61 (CI 3.56, 1229.85) (8) Need for intubation: —(10 unrestrained required intubation compared to 0 restrained)	
Al-Jazaeri et al.;2012; Saudi Arabia [26]	Younger than 13 years old	Retrospective trauma registry review	Unrestrained: 89 Front seat (FS): 41 Back seat (BS): 48		Traumatic brain injury FS: 58.5% BS: 47.9% Facial fractures FS: 4.9% BS: 16.7% C-spine injury FS: 7.3% BS: 6.3% Chest trauma FS: 9.8% BS: 16.7% Abdominal trauma FS: 19.5% BS: 12.5% Upper extremities fracture FS: 19.5% BS: 25% Lower extremities fracture FS: 17.1% BS: 27.1%

CI: Confidence interval; FS: front seat; BS: back seat.
